# Progress in Cancer

**Published:** 1901-06-01

**Authors:** 


### Progress in Cancer.

(*Continued from page* 135.)

With regard to the search for the parasite of cancer, Schüller [3] finds an organism consistently present in all sarcomata and carcinomata. They are three or four times the size of red blood corpuscles, round or oval with a striated appearance. The latter may be due to pores, through which the contained protoplasm may be seen to escape; and empty capsules are found. In hanging-drop preparations flagella-like processes may be recognised, although the organism is never motile. The parasite can be seen in fresh specimens cleared with lavender oil. Stained specimens are of little value. Animal inoculations are too recent to be of any value. The culture medium was the tumour tissue.

But Obt [4] finds that the so-called parasite shows resistance to the action of acids and alkalies similar to red corpuscles. Also that they contain iron. He supposes, therefore, that the epithelial cells devour the degenerated red cells.

After special antiseptic precautions, Maragliano [5] found nine out of 33 cases of cancer showed staphylococci in the blood, without pyrexia or evidence of septicæmia. These nine cases had ulcerated.

The leucocytosis consequent upon digestion has been held by many observers as a diagnostic point in reference to diseases of the abdominal organs. In cancer it is said that this leucocytosis does not occur, and that its absence is diagnostic of that disease. However, Carstairs Douglas,[6] following on the work of Osler, Macrae, and others, shows that out of 11 cases only 54 per cent. gave a positive reaction in regard to this. It is probable, therefore, that Marchetti is right, when he states that in his belief leucocytosis depends upon the digestive and absorptive powers of the stomach. Cancer may or may not have impaired its functions.

Where doubt is thrown upon old points, a new sign in the diagnosis of abdominal cancer is always welcome. Dr. Ebret [7] draws attention to the presence of the B. filiformis in the stomach contents. When this is present in quantity in a fasting stomach, when the contents are moderate (100 cc), then the diagnosis may be affirmed. It may be recognised by its characteristic cotton-like growth in two days on agar agar with 1 per cent. glucose; or its woolly precipitate after three days in acidulated bouillon.

Paul [8] has examined cases of epithelioma of the skin and rodent ulcer in their earliest stages, and finds that epithelioma always begins as "an overgrowth of the epidermis," and it is only after a variable amount of upward growth of a papillomatous type that it buds downwards into the skin and subcutaneous tissues to present its typical malignant structure.

In rodent ulcer, however, "the epidermis is thinned and flattened over the papule." In a typical example of early ulcer the sweat glands lie unaltered beneath the tumour, the rete is unaffected above, while the sebaceous gland is lost in the growth. Paul suggests that rodent ulcer is a slow-glowing carcinoma of the sebaceous glands. Apropos of this, Bursfield [9] gives a case of rodent ulcer occurring in a sebaceous cyst. Paul also gives notes of a case which differed from rodent ulcer while having many of its characteristics, which he considers to have been a slow-growing cancer of the sweat glands—the affections of the skin appendages differing from each other, as the structures of those organs themselves.

Although certain places in the body are looked upon as the frequent sites of cancer, yet many cases are reported from time to time of primary tumours occurring in "unusual" positions.

Thus Nicholas [10] has collected 38 cases of primary cancer of the bronchi, with a predisposition for the right side and for males.

A malignant tumour has appeared simultaneously in both parotid regions, in a case under the care of Dr. Snow.[11] The tumours appeared first during pregnancy, two years previously.

Not many cases are recorded of primary carcinoma of the vermiform appendix, yet Whipham [12] records a case at St. George's Hospital, attended by great thickening of the intima and was of spheroidal-celled type; secondary nodules in the liver and left ovary. In July Rolleston recorded a similar case. In January the same observer [13] records the supervention of primary carcinoma on cirrhosis in the liver. He considers that it may be regarded as an attempt on the part of the liver cells in the direction of compensatory hyperplasia.

Serious attention is being drawn to oöphorectomy in the treatment of inoperable mammary cancer. Stanley Boyd,[14] who so strongly advocates the treatment, gives the results of 54 cases, collected or his own.

He divides them into two groups:—

1. Those in which oöphorectomy seemed to produce a clear and considerable result.

2. Those where no effect was produced, or this effect was doubtful.

Chance and the mere beneficial effect of laparotomy by itself, were not the causal agency in the improvement. Thyroid extract had no, perhaps a detrimental, effect.

The progress of disease is arrested in some cases when not cured, life being thereby prolonged and general health improved.

Nearly 6 per cent. obtained immunity for two years and over. There seems to be no effect upon cancer of other organs, not even of the uterus, and not all mammary cancers. This he is inclined to attribute to some pathological change in the ovarian internal secretion affecting the tissues or growth itself.

Comparing his groups, he finds that the unsuccessful cases were younger on the average (37·2 as compared with 42·5), or had passed the menopause, and the general type was worse. No guide can be found to the prognosis of the operation in the quantity of superficial disease. Secondary visceral lesions are bad, and there is no evidence that they are affected by the treatment.

Any evidence of success appears early, change noticed within a week, and within two to four weeks much of the growth gone.

He advises the operation in cases not very acute; in women over 40, with no visceral or bony lesions, in fair condition, and before the menopause.

Dr. Herman[14] also speaks hopefully of the treatment, but he considers that the combination of thyroid extract with the operation is advantageous, and considers it a risk to leave it out, before the double treatment has been studied. Of his four cases the first, operated on in March 1897, is free of cancer; the second, of July 12th, 1898, is now beginning to show ulceration again, but is not yet so bad as before the operation; the third, an extensive case, aged 44, operated on May 3rd, showed great improvement by September 17th; the fourth case, aged 43, with spheroidal-celled cancer of rapid growth and very painful, operated June 2nd. The nodules disappeared by July 1st, but by February 27th had slightly returned; no pain left. Thyroid extract was given and pushed in all his cases where tolerated.

Lockwood[15] declares himself in favour of the treatment, and Walsham has performed the operation, but too recently for notes to be published.

The effect of Finsen's light rays and the X-rays upon rodent ulcer is being watched with interest. Malcolm Morris and Dore[16] show that the former rays have a stimulating effect and soften the edges. On cessation of the treatment, when the inflammation has taken place, healing ensues with a firm and healthy scar. One case was cured after 10 days' exposure.

Sequeira[16] shows good results with the X-rays. He has at present only tried cases unsuitable to the light treatment, owing to position, &c. Using 10-minute exposure, a few days show a marked improvement, and the result a firm healthy-looking scar. Microscopical preparations show a degeneration of the epithelial cells and an increase of connective tissue. Johnson and Merrill[16] have used the X-rays in carcinoma, and hold that this treatment relieves pain and prolongs life in inoperable cases. They aim at producing an X-ray burn. Low-vacuum tubes are necessary, and it is advised to test each tube before use, as some tubes are much slower than others. The object is to produce a mild inflammation and pigmentation around the diseased tissue, gradually increasing its severity until there is a burn of such depth that it will require six weeks to heal on the normal skin. The treatment is renewed as required. The immediate result is palliative, and beneficial permanent result in a few weeks. The treatment is painless.

Seven out of 16 cases of epithelioma treated by Valdemar Bie[16] with concentrated light have shown no recurrence. After some interval (one case 2½ years) five were improved, three received no benefit; of these, however, two were difficult to reach with the pressure tubes. As Williams[16] points out, the treatment of cancer by rays is only limited to superficial disease. Pure resorcin has been used by Bowen Williams[17] in the treatment of rodent ulcer, with the result that complete healing occurred in two months.

[3] Amer. Jour. Med. Sci., September. [4] Brit. Med. Jour., February 9. [5] Brit. Med. Jour., March 2. [6] Brit. Med. Jour., March 16. [7] Brit. Med. Jour., March 16. [8] Brit. Med. Jour., February 9. [9] Brit. Med. Jour., December 1. [10] Brit. Med. Jour., January 5. [11] Brit. Med. Jour., January 12. [12] Lancet, February 2. [13] Brit. Med. Jour., January 19. [14] Brit. Med. Jour., October 20. [15] Clin. Jour., November 2 [16] Brit. Med. Jour., February 9. [17] Brit. Med. Jour., December 1

